# Comparison of Clinical and Radiographic Outcomes Between Single Symphyseal and Parasymphyseal Implants Versus Two Implant-Assisted Complete Mandibular Overdentures

**DOI:** 10.7759/cureus.69845

**Published:** 2024-09-21

**Authors:** Ahmed E Al-Gazzar, Ahmed El-okl, Mohamed Aboshama, Ahmed F Elhagali, Emad Boriqaa

**Affiliations:** 1 Department of Removable Prosthodontics, Al-Azhar University, Asyut, EGY

**Keywords:** bleeding index, implant overdentures, masticatory efficiency, plaque index, pocket depth, single implant

## Abstract

Purpose: This study aimed to compare the efficacy of single implants in the symphyseal and parasymphyseal regions with that of two implants in complete mandibular overdentures.

Patients and methods: Fifteen completely edentulous male patients (50-65 years) were chosen and randomly grouped into three equal groups (five patients in each) according to the position and number of the received implants to assist mandibular complete overdentures: group I: single median implant; group II: single parasymphyseal implant; and group III: two implants were inserted in the canine area bilaterally. After three months of osseointegration, the lower denture was transformed into an implant-assisted complete mandibular overdenture. Clinical observations were documented on the day of loading, and three, six, and nine months later for each implant. Follow-up cone beam computed tomography scans were performed to assess marginal bone loss on the day of loading, and six and twelve months later. The masticatory efficiency evaluation was conducted one and three months after loading.

Results: The obtained results demonstrated insignificant differences between the three groups concerning marginal bone loss, plaque index, pocket depth, and bleeding index. Regarding the masticatory efficiency, the results revealed a significantly higher masticatory efficiency in group III than in groups I and II.

Conclusion: Using two implants to retain mandibular overdentures is the first choice due to their higher masticatory efficiency than a single implant. However, a single implant may be a viable treatment alternative, especially in cases where there are any limitations that restrict the use of additional implants.

## Introduction

Two-implant overdentures are considered a favorable treatment alternative for completely edentulous individuals, especially those with severe residual ridge resorption. They improve the lower denture's retention and stability, improving the patient's biting force, mastication, satisfaction, and quality of life. They are considered a less expensive option than fixed prostheses. They can prevent further esthetic or phonetic issues in certain patients with an interocclusal greater than 15 mm or loss of lip support [1].

Challenges should be considered, including attaining implant parallelism, high expense, and the impact of different implant angulations on overdenture retention. Hence, developing the single-implant concept was suggested as a viable alternative for stabilizing the mandibular complete denture. It may be a favorable treatment for the geriatric population because of their health and financial status. It is also less invasive and expensive, provides good stability and retention, and improves the quality of life and comfort [2].

A common limitation of a single-implant-assisted mandibular complete overdenture is the increased incidence of denture base midline fractures. To improve its mechanical behavior, the acrylic denture base could be reinforced with a zirconia or Co-Cr framework [3].

Liu et al. found that single implants can bear and dissipate the load to the bone well [4]. Bryant et al. compared one and two implants to retain mandibular overdentures at a five-year follow-up and revealed insignificant differences in patient satisfaction or implant survival rate [2].

The parasymphyseal area has been proposed as a site for implant insertion for single-implant-assisted mandibular complete overdentures because the symphyseal area may not have sufficient bone width or may be anatomically limited, making it difficult to insert an implant in this area [5].

Implant-assisted overdentures can significantly help with ridge preservation. Compared to complete dentures, patients with two implants supporting their lower dentures experienced 2.5 times less bone loss [6]. Peri-implant bone preservation is well-established as essential for implant long-term success. The peri-implant bone level is considered a reliable indicator of how well the jawbone responds to implant surgery and the forces of occlusal loading [7]. Plaque index scores and bleeding on probing indices identify early-stage peri-implantitis, while pocket depth measurements delineate the inflammatory status of peri-implant soft tissues [8,9].

The belief that implant-assisted overdenture will consistently enhance masticatory efficiency is the basis for choosing these dentures over conventional dentures. Therefore, the main justification for treatment with implant-assisted overdenture is frequently cited as the patient's desire for increased masticatory function. In addition, compared to the preimplant state, patient satisfaction with a prosthesis kept with an implant was high, so the masticatory efficiency tests gauged functional restoration [10].

This study aimed to compare the use of single implants in the symphyseal and parasymphyseal, and two implants were inserted in the canine area bilaterally to assist mandibular complete overdentures regarding clinical evaluation, marginal bone loss, and masticatory efficiency. The null hypothesis was that there was no statistically significant difference in clinical evaluation, marginal bone loss, and masticatory efficiency among single symphyseal implants, single parasymphyseal implants, and two-implant-assisted complete mandibular overdentures.

## Materials and methods

This randomized controlled clinical trial was conducted at the clinic of the Department of Prosthodontics at Al-Azhar Dental College, Asyut, Egypt. It was approved by the Institutional Ethical Committee of Al-Azhar Dental College, Asyut, Egypt (approval number: AUAREC20210006-5), ensuring compliance with the Declaration of Helsinki. The study protocol was registered on the ClinicalTrials.gov Protocol Registration and Results System under registration number (NCT06599450). Fifteen completely edentulous male patients (50-65 years) were chosen for this study. According to the limited time frame available for this academic study, the study was conducted over 15 months, with a follow-up period of 12 months after prosthesis delivery.

Eligibility criteria

The specified inclusion criteria were as follows: eligible patients possessed a class I maxillomandibular jaw relationship, normal residual ridge morphology, absence of severe bony undercuts or flabby tissue, and coverage by firm mucoperiosteum. A cone beam computed tomography (CBCT) of the lower arch was made to detect the presence of pathological lesions, remaining roots, or impacted teeth in the arch and evaluate both bone quality and quantity, especially in the area of interest.

Exclusion criteria include patients with current chemotherapy or radiotherapy, bleeding disorders, uncontrolled diabetes, history of drug therapy that interferes with bone resorption or deposition (e.g., the prolonged use of glucocorticoids, antiresorptive medications, selective serotonin reuptake inhibitors, and proton-pump inhibitors), any physical reasons that could affect follow-up, and psychiatric problems, heavy smokers, drug or alcohol addicts, immunocompromised patients, and patients with abnormal jaw relationship, inadequate interarch space, temporomandibular disorders, and parafunctional habits (e.g., bruxing and clenching).

**Figure 1:**
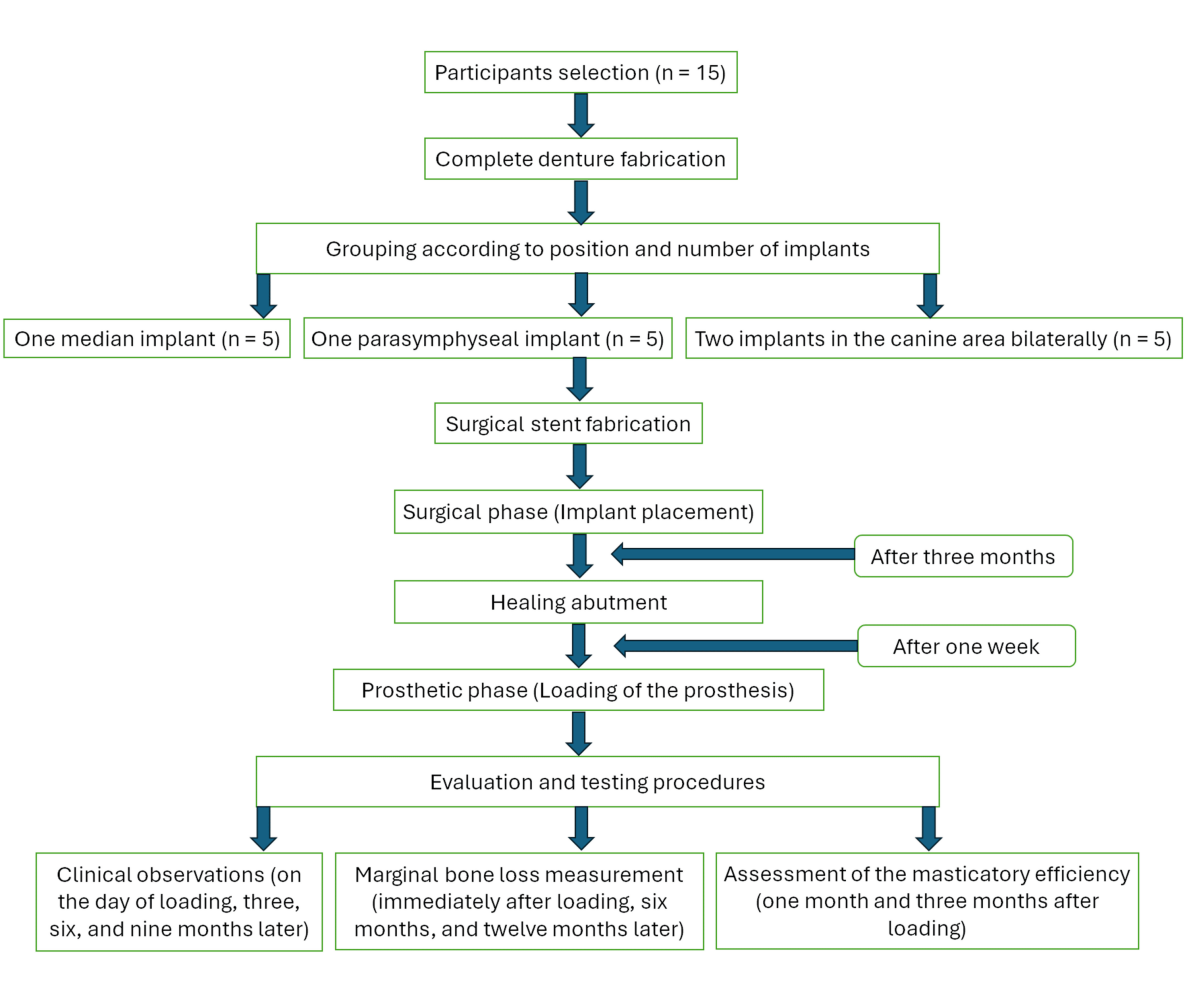
Flow diagram of the study

Each individual signed an informed consent form, which the ethics committee approved, after explaining the treatment plan before initiating treatment. The study course followed the flowchart depicted in Figure 1.

For each individual, a complete denture was fabricated from heat-polymerized acrylic resin denture base material, anatomic maxillary teeth, and nonanatomic mandibular teeth (Acrostone, Cairo, Egypt) according to the manufacturer's instructions with a lingualized occlusal scheme using a facebow and semiadjustable articulator (Bio-Art, Brazil). The individuals were randomly grouped according to position and number of implants received to assist mandibular complete overdenture into three equal groups, each consisting of five patients (n = 5): group I received one median implant, group II received one parasymphyseal implant, and group III received two implants in the canine area bilaterally. This stratified random sampling approach ensured a balanced distribution of patients across the three implant groups, allowing for an adequate comparison of treatment outcomes.

**Figure 2:**
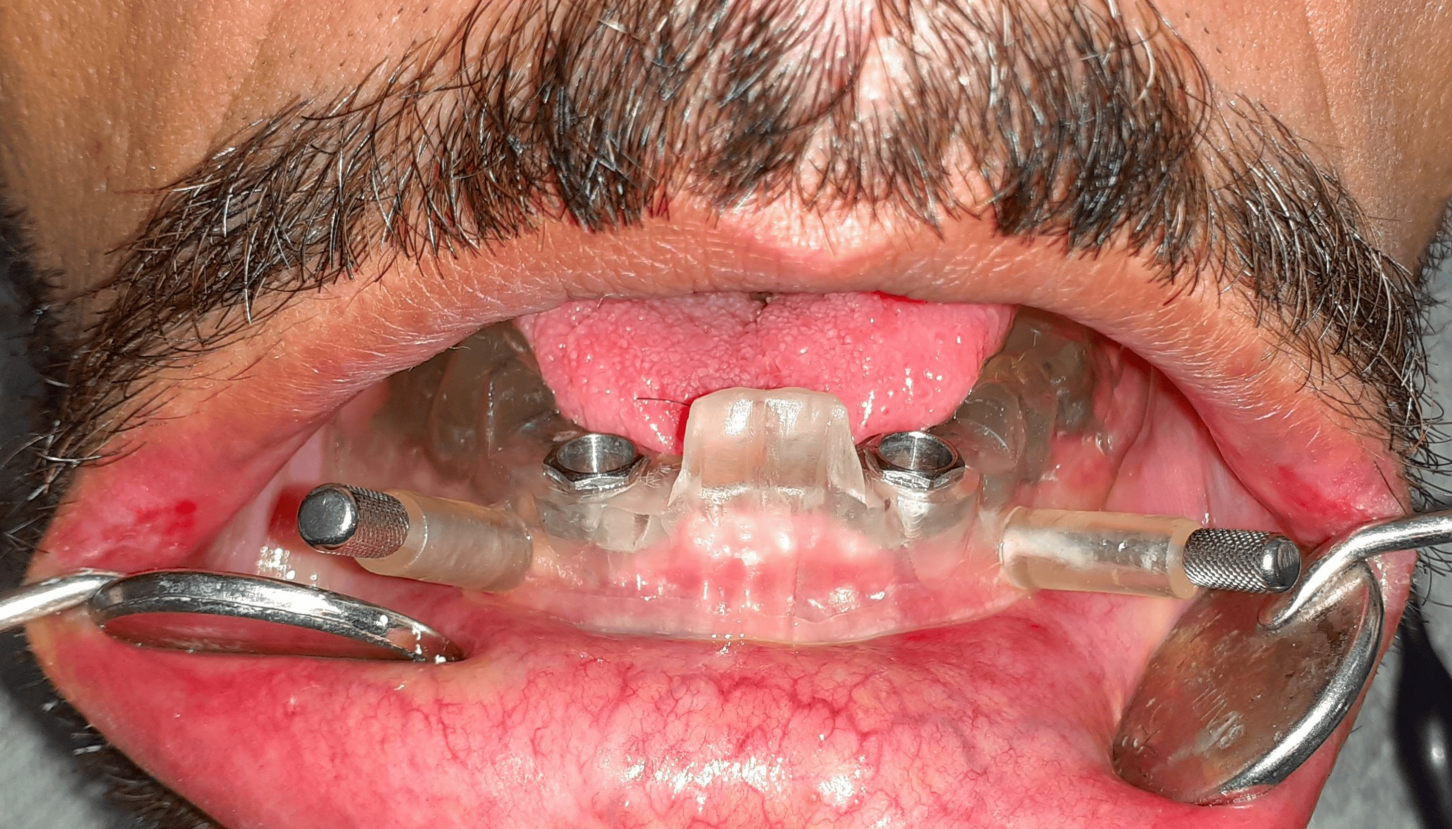
A mucosa-supported surgical guide

The lower dentures were duplicated into a transparent self-cure acrylic resin model; holes were drilled into the model and then filled with gutta-percha pieces to serve as radiopaque markers for creating the radiographic template. The dual-scan technique was used where the radiographic template and the patients' plaster casts were scanned by CBCT at 10 mA and 85 kV (denture scan mode). Then, the patients were asked to wear the radiographic template and the upper denture for another scan using CBCT at 15 mA and 85 kV, with an exposure time of 18 seconds, a voxel size of 0.2 mm, and a field of view of 7.5 cm × 14.5 cm × 14.5 cm. A CBCT machine (Planmeca ProMax 3D, Planmeca, Finland) was used for scanning. The Digital Imaging and Communications in Medicine files were uploaded via Ethernet to the specialized computer software for reconstruction (Blue Sky Plan, version 4.13, Blue Sky Bio, Libertyville, IL). Subsequently, a virtual implant simulation and the design of the virtual template were carried out and saved as a Standard Tessellation Language file. The surgical stent was then printed using a 3D printer from the photosensitive polymerized material (Perfactory® Clear Guide, EnvisionTEC, Inc., Germany) [11]. All patients received a 3.5-diameter, 10-length dental implant fixture (Is-II active, Neobiotech Implant System, Wonju-si, Korea) using flapless implant surgery and left three months for osseointegration before loading dental implants with a mandibular overdenture (Figure 2).

**Figure 3:**
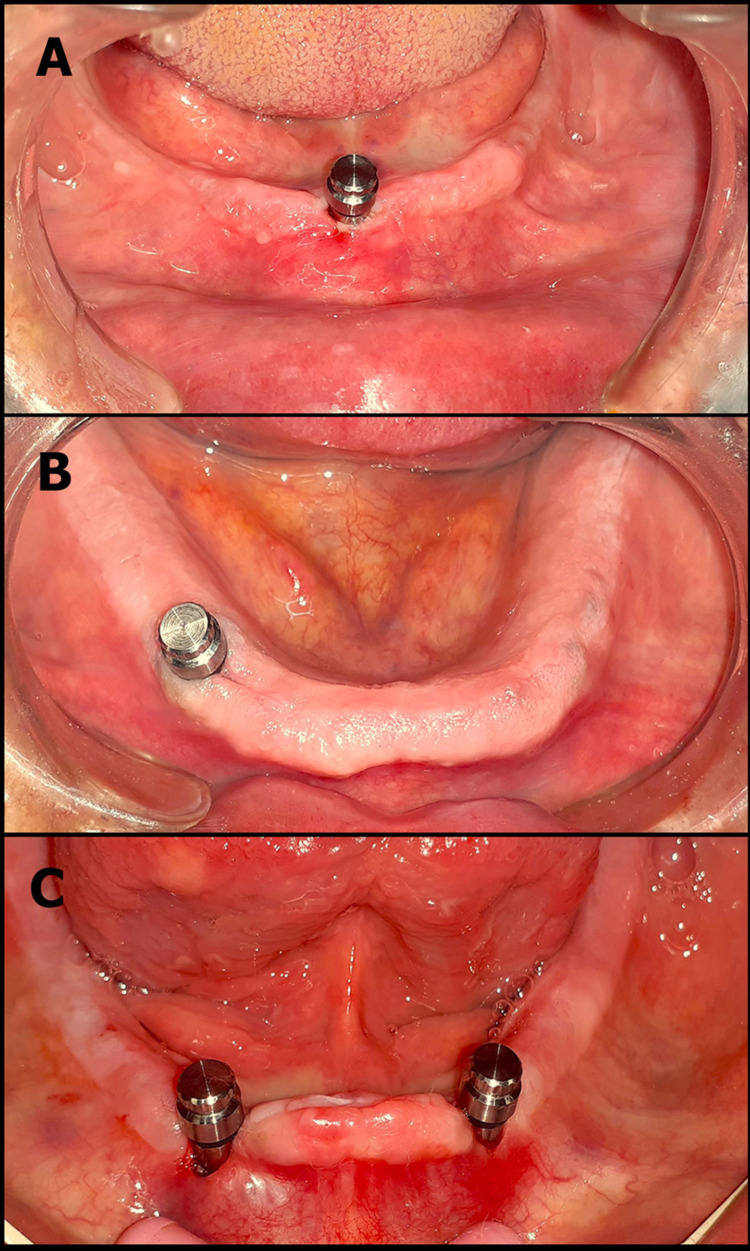
The metal housing is in its place above the ball abutment. (A) One median implant. (B) One parasymphyseal implant. (C) Two implants in the intraforaminal distance

After three months, the implant osseointegration was evaluated using periapical film and intraoral examination before loading. In the second stage, the surgical guide relocates the implant site. The implant was exposed, and the healing cap was removed using a hex driver and replaced with a healing abutment, which was left in place for a week to facilitate the gingival tissue healing and then replaced with the ball and socket attachment. According to manufacturer instructions (Neobiotech Implant System), relief was made on the impression surface of the lower denture at the implant area using an acrylic bur. The metal housing has a temporary processing rubber O-ring to prevent the engagement of any undercuts with excess acrylic resin. For pickup of the housing, it was positioned on the implant abutment (Figure 3). After ensuring the proper seating of the denture, the relieved area was filled with chemical-cured acrylic resin and placed in the patient's mouth, which was instructed to bite gently while the acrylic resin was being set. After the resin was set, the denture was removed, and the processing rubber O-ring was replaced with a clinical rubber O-ring; then, any excess resin was trimmed. The retention and occlusion were checked intraorally, and final adjustments were made.

Clinical observations

The presence and amount of plaque were determined at the four sites around the implant (mesial, distal, buccal, and lingual) and scored from 0 to 3: grade 0: no plaque by probing; grade 1: film of plaque detected by probing; grade 2: visible plaque with the naked eye; and grade 3: abundant soft material in the sulcus, mucosal margin, and adjacent implant surface. The mean of the four surface readings was considered the plaque index for this group at the chosen times [8].

The pocket depth was measured by inserting a graduated plastic periodontal probe between the oral sulcular epithelium and the implant with minimal pressure at the midpoint of the four surfaces. The distance from the bottom of the sulcus to the mucosal margin was measured and recorded to the nearest millimeter; the mean of four readings was considered the pocket depth for this group at the chosen time [9].

The bleeding index was assessed for the four surfaces while measuring the pocket depth if the bleeding was visible after probing within 20 seconds. It is typically scored as 0 (no bleeding), 1 (bleeding point), 2 (single line of blood), or 3 (heavy or profuse bleeding). The mean of four readings was considered the bleeding index for this group at the chosen time [9]. The clinical evaluation was documented at follow-up visits on the day of loading and three, six, and nine months later for each implant.

Marginal bone loss measurement

**Figure 4:**
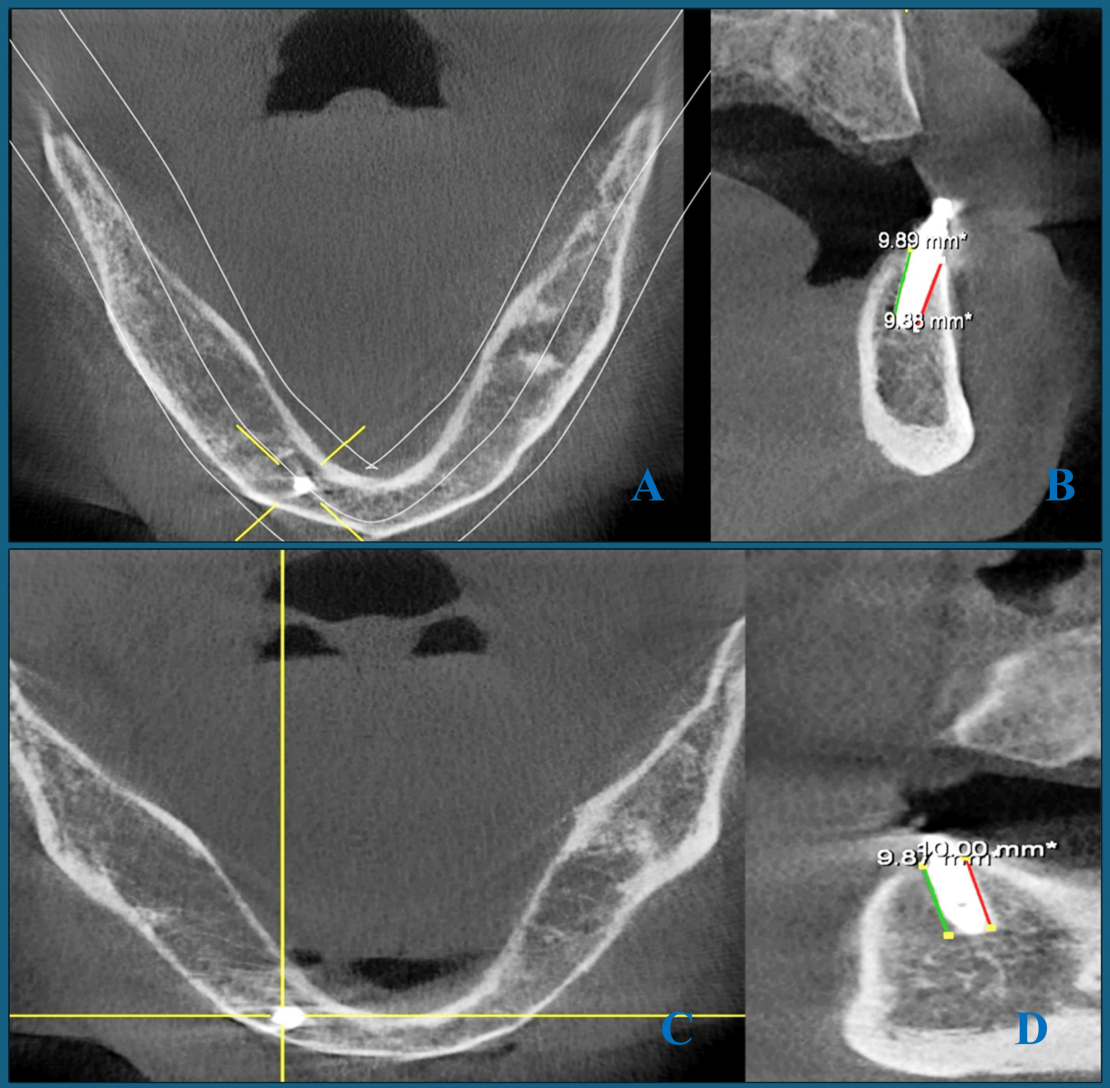
(A) Axial CBCT image displaying the panoramic reconstructed curve. (B) Reconstructed cross-sectional image displaying measurements of the buccal and lingual bone levels surrounding the implant. (C) Axial CBCT image displaying the alignment of the coronal section to pass through the implant. (D) Coronal image displaying measurements of the distal and mesial bone levels surrounding the implant CBCT: cone beam computed tomography

A CBCT was performed immediately after loading and then at 6 and 12 months later to assess the marginal bone loss. The cross-sectional reconstructed image measured buccal and lingual bone levels around implants, and the coronal image measured distal and mesial bone levels around implants (Figure 4). The measuring line extended from the most coronal point of implant-bone attachment to the implant end (apex), passing tangential to the flutes of the implant (but not touching the implant body). Calculating average bone height involves dividing the sum of buccal, lingual, mesial, and distal bone height by 4. The real bone loss can be calculated by subtraction from the implant length [11].

Assessment of the masticatory efficiency

The masticatory efficiency was assessed when the patient ate three different types of food (carrot, apple, and banana) with varying degrees of hardness, cut into standardized pieces (1 cm cube each). The collected data included the following measurements: the chewing stroke count before the first swallow, the chewing stroke count until the oral cavity is empty of food, the count of swallows until the oral cavity is empty of food, the time estimated in seconds until the first swallow, and the time estimated in seconds until the oral cavity is empty of food. The evaluation was documented at follow-up visits one and three months after the overdenture insertion [10].

Statistical analysis

Statistical analysis was done using the IBM® SPSS® Statistics software, version 26 (IBM Corp., Armonk, NY). The mean and standard deviation (SD) were estimated for each patient, and a paired t-test was utilized to compare two follow-ups in the same group. A one-way repeated analysis of variance (ANOVA) was performed to compare more than two follow-ups in the same group, followed by Tukey's post hoc test for pairwise comparisons (adjusted Bonferroni) when the ANOVA test was significant. A one-way ANOVA was used to compare the tested groups, followed by Tukey's post hoc test for pairwise comparisons when the ANOVA test was significant. The significance level was set at p ≤ 0.05 and a 95% confidence interval.

## Results

All chosen patients remained in the trial until its conclusion; none experienced postoperative problems.

Clinical observations

**Figure 5:**
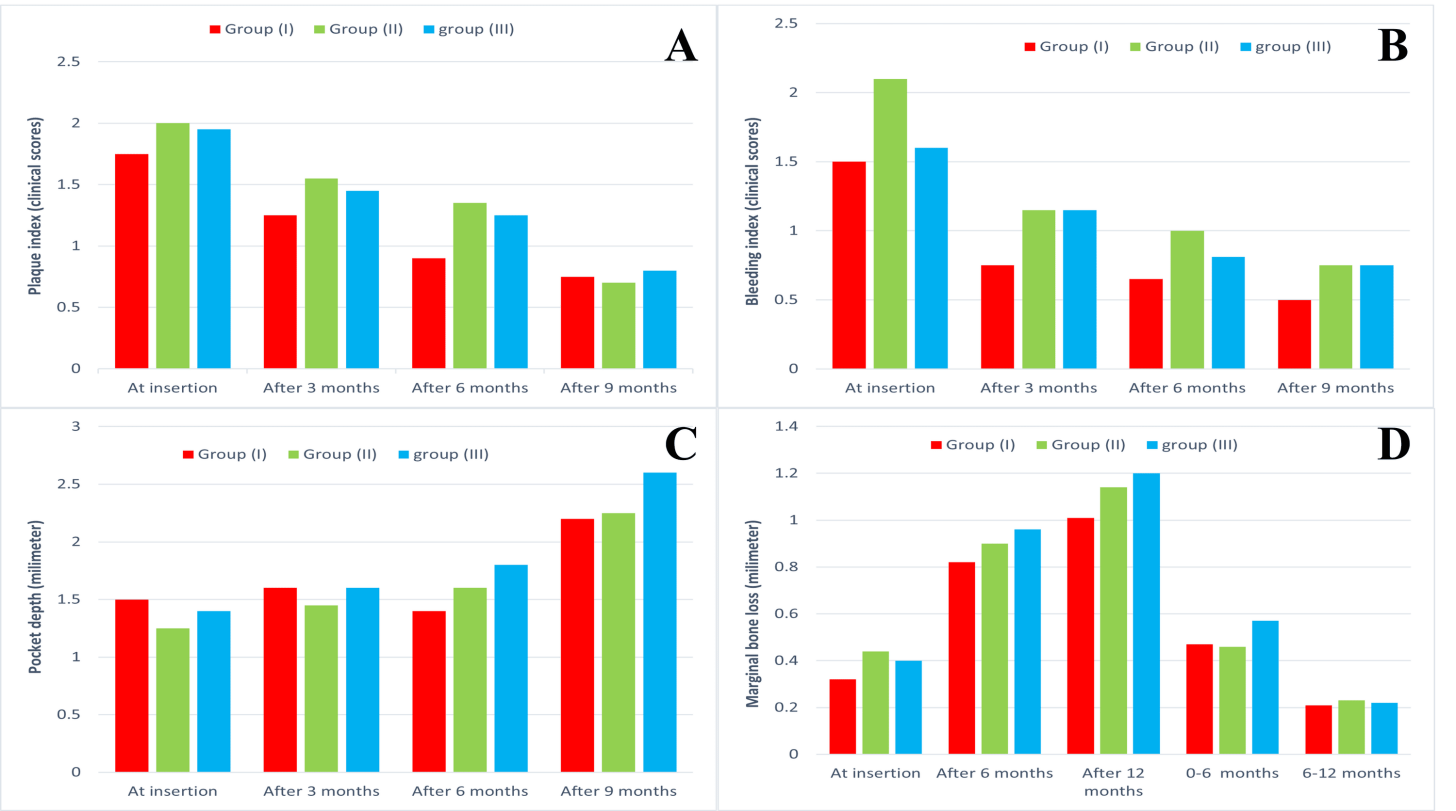
Intergroup comparison of (A) plaque index, (B) bleeding index, (C) pocket depth, and (D) marginal bone loss The results are presented as mean value for each group

Plaque index, pocket depth, and bleeding index were assessed on the buccal, lingual, mesial, and distal aspects of each implant, and mean values were calculated on the day of loading, three months, six months, and nine months after loading. Table 1 and Figure 5 summarize the mean ± SD scores of plaque and bleeding indices and pocket depth.

**Table 1:** Comparison of plaque index, bleeding index, pocket depth, and marginal bone loss between successive time intervals (two follow-ups by paired t-test, and three or four follow-ups by one-way repeated ANOVA test followed by Tukey’s post hoc test for pairwise comparisons when the ANOVA test was significant), and between groups (intergroup comparison using one-way ANOVA test) The results are presented as mean ± SD Different letters in the same column show significance (p < 0.05) p: probability level; *: significant; p < 0.05: significant; p < 0.001: highly significant; ns: nonsignificant (p > 0.05); ANOVA: analysis of variance; SD: standard deviation

Peri-implant assessment	Time interval	Group I	Group II	Group III	p value
Plaque index	At insertion	1.75^a ^± 0.25	2^a ^± 0.17	1.95^a ^± 0.20	0.191
	After three months	1.25^a,b ^± 0.30	1.55^a ^± 0.32	1.45^a,b ^± 0.54	0.512
	After six months	0.9^b,c ^± 0.28	1.35^a ^± 0.28	1.25^a,b ^± 0.39	0.113
	After nine months	0.75^c ^± 0.17	0.70^b ^± 0.27	0.80^b ^± 0.20	0.783
	p value	0.000^*^	0.000^*^	0.000^*^	-
Bleeding index	At insertion	1.5^a ^± 0.17	2.1^a ^± 0.41	1.6^a ^± 0.51	0.074
	After three months	0.75^b ^± 0.17	1.15^b^ ± 0.28	1.15^a,b ^± 0.45	0.123
	After six months	0.65^b ^± 0.28	1^b ^± 0.17	0.81^b ^± 0.52	0.338
	After nine months	0.50^b ^± 0.17	0.75^b ^± 0.25	0.75^b ^± 0.30	0.230
	p value	0.000^*^	0.000^*^	0.001^*^	-
Pocket depth	At insertion	1.5^a,b ^± 0.39	1.25^b ^± 0.39	1.4^b ^± 0.51	0.673
	After three months	1.6^a,b^ ± 0.67	1.45^a,b ^± 0.44	1.6^a,b ^± 0.38	0.860
	After six months	1.4^b ^± 0.51	1.6^a,b ^± 0.41	1.8^a,b ^± 0.20	0.326
	After nine months	2.2^a ^± 0.20	2.25^a ^± 0.50	2.6^a ^± 0.62	0.384
	p value	0.014^*^	0.005^*^	0.001^*^	-
Marginal bone loss	At insertion	0.32^c ^± 0.11	0.44^c^ ± 0.12	0.40^c^ ± 0.10	0.285
	After six months	0.82^b^ ± 0.12	0.90^b^ ± 0.08	0.96^b^ ± 0.16	0.268
	After 12 months	1.01^a^ ± 0.15	1.14^a^ ± 0.11	1.20^a^ ± 0.09	0.070
	p value	0.000^*^	0.001^*^	0.000^*^	-
	0-6 months	0.47^a^ ± 0.07	0.46^a^ ± 0.13	0.57^a^ ± 0.15	0.318
	6-12 months	0.21^b^ ± 0.06	0.23^b^ ± 0.03	0.22^b^ ± 0.10	0.839
	p value	0.002^*^	0.008^*^	0.047^*^	-

A one-way repeated ANOVA indicated a significant decrease in the plaque and bleeding indexes within each group throughout the follow-up period. In contrast, Table 1 and Figure 5 showed a significant increase in pocket depth within each group.

When the plaque index, pocket depth, and bleeding index were compared between the groups using one-way ANOVA tests, there was no statistically significant difference between the three groups, as shown in Table 1 and Figure 5.

Marginal bone loss

Marginal bone loss was measured on the buccal, lingual, mesial, and distal aspects of each implant, and the mean values were calculated on the day of loading, and 6 and 12 months after loading. A summary of the mean ± SD scores of marginal bone loss is presented in Table 1 and Figure 5.

A one-way repeated ANOVA indicated a significant increase in marginal bone loss within each group throughout the follow-up period. However, using one-way ANOVA tests, an intergroup comparison of marginal bone loss found no statistically significant difference between the three groups, as shown in Table 1 and Figure 5.

Comparison of interval periods (0-6 and 6-12 months) within each group using a paired t-test revealed a significant increase in marginal bone loss in the 0-6 month interval compared to the 6-12 month interval. In the intergroup comparison, one-way ANOVA tests showed no statistically significant difference between the three groups, as shown in Table 1 and Figure 5.

The masticatory efficiency

**Figure 6:**
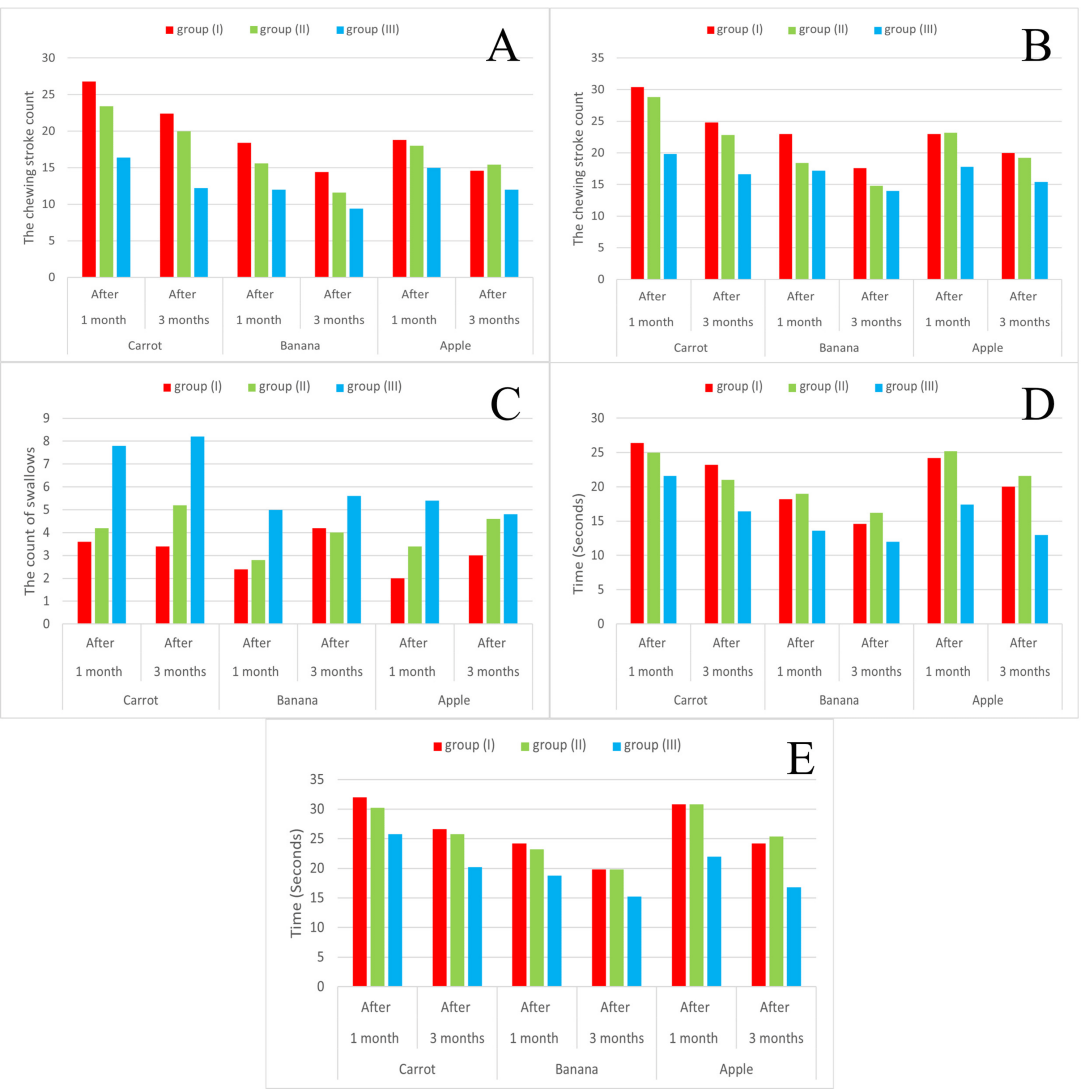
Intergroup comparison of the masticatory efficiency between the groups for three different types of food (carrot, banana, and apple) regarding the chewing stroke count before the first swallow (A), the chewing stroke count until the oral cavity empties food (B), the count of swallows until the oral cavity empties food (C), the time estimated in seconds until the first swallow (D), and the time estimated in seconds until the oral cavity is empty of food (E) The results are presented as mean value for each group

A summary of the mean ± SD scores of masticatory efficiencies is presented in Table 2 and Figure 6.

**Table 2:** Comparison of the masticatory efficiency between successive time intervals (two follow-ups by paired t-test) and between the groups (intergroup comparison using one-way ANOVA test followed by Tukey's post hoc test for pairwise comparisons when the ANOVA test was significant for three different types of food: carrot, apple, and banana) The results are presented as mean ± SD Different letters in the same column show significance (p < 0.05) p: probability level; *: significant; p < 0.05: significant; p < 0.001: highly significant; ns: nonsignificant (p > 0.05); A: the chewing stroke count before the first swallow; B: the chewing stroke count until the oral cavity is empty of food; C: the count of swallows until the oral cavity is empty of food; D: the time estimated in seconds until the first swallow; E: the time estimated in seconds until the oral cavity is empty of food; ANOVA: analysis of variance; SD: standard deviation

Masticatory efficiency measure	Group	Carrot			Banana			Apple		
		After one month	After three months	p value	After one month	After three months	p value	After one month	After three months	p value
A	Group I	26.8^a^ ± 2.94	22.4^a^ ± 2.3	0.035^*^	18.4^a^ ± 1.81	14.4^a^ ± 1.14	0.028^*^	18.8^a^ ± 1.78	14.6^a^ ± 1.51	0.012*
	Group II	23.4^a^ ± 2.07	20^a^ ± 1.58	0.030^*^	15.6^b^ ± 1.51	11.6^b^ ± 1.14	0.016^*^	18^a^ ± 0.7	15.4^a^ ± 1.14	0.003*
	Group III	16.4^b^ ± 2.19	12.2^b^ ± 0.83	0.008^*^	12^c^ ± 0.7	9.4^c^ ± 1.34	0.007^*^	15^b^ ± 1.22	12^b^ ± 0.7	0.013*
	p value	0.000^*^	0.000^*^	-	0.000^*^	0.000^*^	-	0.002^*^	0.002^*^	-
B	Group I	30.4^a^ ± 1.67	24.8^a^ ± 2.38	0.004^*^	23^a^ ± 2.23	17.6^a^ ± 1.81	0.030^*^	23^a^ ± 0.7	20^a^ ± 1.87	0.018*
	Group II	28.8^a^ ± 2.86	22.8^a^ ± 1.48	0.034^*^	18.4^b^ ± 1.51	14.8^b^ ± 0.83	0.009^*^	23.2^a^ ± 1.48	19.2^a^ ± 0.83	0.011*
	Group III	19.8^b^ ± 2.86	16.6^b^ ± 1.14	0.020^*^	17.2^b^ ± 1.64	14^b^ ± 1	0.001^*^	17.8^b^ ± 0.83	15.4^b^ ± 1.14	0.009*
	p value	0.000^*^	0.000^*^	-	0.001^*^	0.002^*^	-	0.000^*^	0.000^*^	-
C	Group I	3.6^b^ ± 1.51	3.4^c^ ± 1.14	0.847	2.4^b^ ± 1.14	4.2^a^ ± 1.92	0.137	2^b^ ± 0.7	3 ± 1.22	0.189
	Group II	4.2^b^ ± 1.3	5.2^b^ ± 0.83	0.298	2.8^b^ ± 1.48	4^a^ ± 1	0.033*	3.4^b^ ± 1.14	4.6 ± 1.51	0.145
	Group III	7.8^a^ ± 2.38	8.2^a^ ± 0.83	0.717	5^a^ ± 0.7	5.6^a^ ± 1.14	0.468	5.4^a^ ± 1.14	4.8 ± 1.3	0.305
	p value	0.006*	0.000*	-	0.008^*^	0.192	-	0.001^*^	0.111	-
D	Group I	26.4^a^ ± 1.14	23.2^a^ ± 1.92	0.045^*^	18.2^a^ ± 1.92	14.6^b^ ± 1.14	0.014^*^	24.2^a^ ± 0.83	20^a^ ± 2.23	0.003*
	Group II	25^a^ ± 1.87	21^a^ ± 1.22	0.007^*^	19^a^ ± 1.22	16.2^a^ ± 0.83	0.019^*^	25.2^a^ ± 0.83	21.6^a^ ± 0.89	0.002*
	Group III	21.6^b^ ± 2.07	16.4^b^ ± 1.51	0.024^*^	13.6^b^ ± 1.14	12^c^ ± 0.7	0.035^*^	17.4^b^ ± 1.81	13^b^ ± 0.7	0.001*
	p value	0.003^*^	0.000^*^	-	0.000^*^	0.000^*^	-	0.000^*^	0.000^*^	-
E	Group I	32^a^ ± 1.58	26.6^a^ ± 1.51	0.012^*^	24.2^a^ ± 1.48	19.8^a^ ± 1.3	0.001^*^	30.8^a^ ± 3.11	24.2^a^ ± 2.28	0.002^*^
	Group II	30.2^a^ ± 1.92	25.8^a^ ± 1.3	0.009^*^	23.2^a^ ± 0.83	19.8^a^ ± 0.83	0.005^*^	30.8^a^ ± 2.38	25.4^a^ ± 1.14	0.009^*^
	Group III	25.8^b^ ± 2.16	20.2^b^ ± 1.92	0.033^*^	18.8^b^ ± 1.48	15.2^b^ ± 0.83	0.011^*^	22^b^ ± 0.7	16.8^b^ ± 0.83	0.001^*^
	p value	0.001^*^	0.000^*^	-	0.000^*^	0.000^*^	-	0.000^*^	0.000^*^	-

For all groups, the paired sample t-test of the masticatory efficiency mean values revealed a significant increase (after three-month follow-up periods) when chewing the three test foods compared to one-month follow-up periods, except in the count of swallows until the oral cavity was empty of food, where the difference was nonsignificant, as shown in Table 2 and Figure 6.

On intergroup comparison of the masticatory efficiency using one-way ANOVA tests, it was found that there was a significant increase in the masticatory efficiency at the two different follow-up periods for group III during chewing of the three test foods compared to that of groups I and II, with a nonsignificant difference between groups I and II, as shown in Table 2 and Figure 6.

## Discussion

A two-implant-assisted mandibular overdenture was recommended to edentulous individuals due to its better retention, stability, and greater satisfaction than the complete denture [1]. The single-implant-assisted mandibular overdenture concept gives another choice for elderly populations to reduce the time and cost of treatment, especially those with low economic status in developing countries [2]. The parasymphyseal area has been suggested for implant insertion for a single-implant-assisted mandibular overdenture due to anatomical limitations on the symphyseal area and the potential risk factors for mandibular arch fracture, especially in older patients [5].

This study used computer-guided surgery, which includes virtual implant planning using computer tomography images and the creation of an accurate digital surgical stent that enables the surgeon to insert implants into predetermined positions precisely [12]. The flapless surgical protocol eliminates the need for surgical flap elevation and subsequent exposure of the underlying bone to insert the implant. This leads to improvements in patient comfort and acceptance, a reduction in soft tissue loss that heals more quickly with minimal problems, a decrease in inflammation and pain, reduced surgical technique time, improved recovery, and helps to avoid angulation of the implants, which can affect the retention of overdentures [13,14].

CBCT imaging can provide a three-dimensional image of the bone. This image offers detailed information about the bone's location, width, volume, and degree of mineralization. Additionally, CBCT can accurately assess bone loss around the implant in all directions, including buccal, lingual, mesial, and distal aspects. It can also diagnose bone defects such as fenestrations and dehiscence [15]. Currently, ball and socket are the most used attachments due to their simplicity. They need only chairside work, do not require a large prosthetic space, allow hinge and rotation dislodgements, and have versatility and applicability in several situations with no need for new denture fabrication [16].

Regarding plaque and bleeding indices, the findings of this study demonstrated a significant decrease during the follow-up periods in each group, with an insignificant difference between the tested groups. This could be attributed to the patient's efforts to maintain a high level of oral hygiene and routine hygienic recall visits, the unsplinted design, the smooth surface of the ball attachments, and the implant's anterior location, enabling easy access and execution of the cleaning and oral hygiene measures, as reported in previous studies [17,18].

The present study showed a negligible difference in pocket depths between all groups during the follow-up periods, which revealed that probing depth is not influenced by the number and location of implants that assist mandibular overdenture. On the other hand, an increase in the pocket depth within each group is obtained through the follow-up period. These findings were within acceptable values and may be associated with bone resorption during the first year after implant placement. These results align with the findings reported by Hauck et al., who also documented increasing in the probing depth after a one-year follow-up period due to the marginal bone loss that occurred during the maturation of bone after implant placement and the adaptation of bone to withstand functional forces [18]. However, the lack of significant difference between some observation periods could be attributed to the gingival recession that may have occurred around the implants.

After three months, a statistically significant increase in the masticatory efficiency was observed in all test groups. This matches the previous studies, which reported that the neuromuscular adaptation to the new denture was achieved, and patients usually function much better with their prosthesis after three months, irrespective of the prosthesis type, and become more experienced in dealing with it functionally [19,20]. However, the difference in the count of swallows until the oral cavity is empty of food during chewing of the three test foods between the follow-up periods was negligible. This suggests that the count of swallows until the oral cavity is empty of food has less sensitivity to determining masticatory efficiency [10].

A significant increase in masticatory efficiency at the two different follow-up periods for group III compared to that of groups I and II was obtained, which could be attributed to the fact that two implants provide more retention, stability, and support, which allows for more efficient chewing and improvement of oral function [21]. These findings agreed with another previous study, which compared masticatory efficiency and patient satisfaction for single- and two-implant-assisted overdentures in the same patient and reported that the better masticatory efficiency of two implants was significant; the patients did not find much difference between one- and two-implant-assisted overdentures [22]. However, other studies have demonstrated that a single-implant-assisted mandibular overdenture can produce greater enhancements in masticatory efficiency and patient satisfaction compared to using two implants. It also has the advantages of short treatment time, less tissue damage, accelerated healing, and reduced cost [23,24].

The obtained results demonstrated insignificant differences in marginal bone loss between all test groups. These findings agreed with previous studies, which reported that the overdenture can rotate laterally over the single implant without an increase in strain, which does not occur with two implants [4,18,25]. On the other hand, these findings disagree with those of Maryod et al., who reported a significant reduction in both posterior residual ridge and resorption marginal bone loss in mandibular implant-retained overdentures with increasing the number of implants [26].

A higher marginal bone loss was observed during the 0-6 month than the 6-12 month interval for all test groups. These results agreed with other previous studies, which reported that marginal bone loss occurs mainly in the first period after loading, followed by stabilization or even slight marginal bone gain [27,28]. This could be explained by the fact that, after implant placement and loading, the surrounding bone undergoes an initial remodeling phase to adapt to the new biomechanical environment. This typically results in some bone resorption in the first few months. After this initial peak, the bone tends to stabilize, and resorption slows down significantly [29].

## Conclusions

Within the limitations of this study (including the patient number and study intervals), the following conclusion can be stated: oral rehabilitation by a single-implant-assisted complete mandibular overdenture may be a viable treatment alternative for edentulous mandibles. This treatment modality's importance increases in cases with any limitation, restricting the use of more implants. Two implants retained mandibular overdentures are the first choice due to their higher masticatory efficiency than a single implant.
